# The relationship between epigenetic biomarkers and the risk of diabetes and cancer: a machine learning modeling approach

**DOI:** 10.3389/fpubh.2025.1509458

**Published:** 2025-03-21

**Authors:** Shiqi Zhang, Jianan Jin, Benfeng Xu, Qi Zheng, Haibo Mou

**Affiliations:** ^1^Department of Oncology, Shulan (Hangzhou) Hospital, Affiliated to Zhejiang Shuren University Shulan International Medical College, Hangzhou, Zhejiang, China; ^2^Graduate School, Zhejiang Chinese Medical University, Hangzhou, Zhejiang, China; ^3^State Key Laboratory for Diagnosis and Treatment of Infectious Diseases, National Clinical Research Center for Infectious Diseases, National Medical Center for Infectious Diseases, Collaborative Innovation Center for Diagnosis and Treatment of Infectious Diseases, The First Affiliated Hospital, Zhejiang University School of Medicine, Hangzhou, China

**Keywords:** epigenetic biomarkers, epigenetic clocks, epigenetic age acceleration, diabetes, cancer, machine learning

## Abstract

**Introduction:**

Epigenetic biomarkers are molecular indicators of epigenetic changes, and some studies have suggested that these biomarkers have predictive power for disease risk. This study aims to analyze the relationship between 30 epigenetic biomarkers and the risk of diabetes and cancer using machine learning modeling.

**Methods:**

The data for this study were sourced from the NHANES database, which includes DNA methylation arrays and epigenetic biomarker datasets. Nine machine learning algorithms were used to build models: AdaBoost, GBM, KNN, lightGBM, MLP, RF, SVM, XGBoost, and logistics. Model stability was evaluated using metrics such as Accuracy, MCC, and Sensitivity. The performance and decision-making ability of the models were displayed using ROC curves and DCA curves, while SHAP values were used to visualize the importance of each epigenetic biomarker.

**Results:**

Epigenetic age acceleration was strongly associated with cancer risk but had a weaker relationship with diabetes. In the diabetes model, the top three contributing features were logA1Mort, family income-to-poverty ratio, and marital status. In the cancer model, the top three contributing features were gender, non-Hispanic White ethnicity, and PACKYRSMort.

**Conclusion:**

Our study identified the relationship between epigenetic biomarkers and the risk of diabetes and cancer, and used machine learning techniques to analyze the contributions of various epigenetic biomarkers to disease risk.

## 1 Introduction

Diabetes and cancer are major challenges in the field of global public health, with the incidence of these diseases continuing to rise (1–3), making it urgent to develop effective early detection signals and risk prediction tools.

Epigenetic biomarkers, particularly DNA methylation, are an important direction for studying the mechanisms underlying these diseases. DNA methylation is a process in which methyl groups are added to DNA, leading to changes in gene function, such as the silencing of tumor suppressor genes, activation of oncogenes, and abnormalities in promoter regions (4–7). These changes are closely linked to the development of various chronic diseases (8–10). However, existing studies have mostly been limited to a small number of epigenetic biomarkers and lack in-depth exploration of the specific relationships between these biomarkers and diseases in different contexts. Epigenetic age clocks are constructed based on specific DNA methylation sites (CpG sites) and can accurately predict an individual’s “biological age,” which refers to the degree of aging of the body compared to its chronological age. By measuring different DNA methylation patterns, researchers have found that epigenetic age is not only highly correlated with chronological age (11), but its acceleration (the difference between epigenetic age and chronological age) is considered a potential biomarker for aging and various diseases (12). For example, accelerated epigenetic age has been found to be closely associated with several age-related diseases, such as diabetes, cancer, cardiovascular diseases, and others (8, 13, 14).

In this study, we present 30 epigenetic biomarkers that have shown potential associations with diabetes and cancer in research across multiple fields. We expanded the selection scope and considered the biological significance of these biomarkers in the context of various diseases. These include epigenetic clocks (such as HorvathAge, LinAge, PhenoAge, WeidnerAge, etc.), lifespan and mortality-related biomarkers (such as GrimAgeMort, ADMMort, B2MMort, GDF15Mort, etc.), immune system biomarkers (such as CRPMor, CD8TPP, CD4TPP, NKcel, Bcell, etc.), and metabolism-related biomarkers (such as logA1CMort, LeptinMort, PACKYRSMort), which fall into three major categories of epigenetic material. For example, biomarkers like HorvathAge and GrimAge are closely associated with the aging process and the onset of various chronic diseases. LinAge, a biomarker associated with an individual’s age, has demonstrated a strong correlation with biological aging (15). Meanwhile, biomarkers such as LeptinMort and PACKYRSMort, which are linked to metabolic processes, smoking behaviors, and nutrition, have been confirmed in multiple studies to be associated with an increased risk of diabetes (16, 17). Additionally, GDF15Mort is related to age-associated mitochondrial dysfunction (18). Therefore, selecting these biomarkers as the focus of this study aims to explore their predictive ability and underlying mechanisms in diabetes and cancer.

Machine learning (ML), as an emerging artificial intelligence technology, offers advantages over traditional statistical analyses, such as higher predictive accuracy and the ability to maintain high prediction performance even with missing data. It is capable of making accurate predictions by utilizing data from various sources. The application of machine learning techniques in the medical field for disease diagnosis and prediction can significantly contribute to disease prevention and treatment (19, 20). In recent years, machine learning has been applied across various fields of clinical disease diagnosis. For example, studies have widely applied machine learning algorithms in the monitoring of tumors and cardiovascular diseases (21). Other research has used machine learning to analyze regularly collected electronic health record data and construct predictive models for the progression from prediabetes to type 2 diabetes (22).

The core objective of this study is to explore the relationship between different epigenetic age clocks and diabetes and cancer, analyzing the potential role of accelerated epigenetic age in these diseases. Secondly, machine learning techniques were used to construct risk assessment models for diabetes and cancer based on epigenetic biomarkers, and their predictive effectiveness in clinical settings was validated. Lastly, by combining clinical features with epigenetic biomarkers, the study aims to optimize risk prediction models, providing a basis for early disease screening and personalized intervention. Nine machine learning algorithms were selected to build the models, and model evaluations and comparisons were conducted to identify the best model for predicting cancer and diabetes risks. Thirty epigenetic biomarkers were used to predict the risk of diabetes and cancer and visualized for analysis. The goal is to assess the value of each epigenetic biomarker in predicting the occurrence of diabetes and cancer, especially in the early screening and prediction of these diseases. Epigenetics will undoubtedly help unravel more mysteries about aging and its associated diseases, offering strong support for the implementation of precision medicine.

## 2 Methods

### 2.1 Data sources and study population

The epigenetic biomarker data in this study are based on the DNA methylation array and epigenetic biomarker datasets from the NHANES database, an epidemiological survey program conducted by the National Center for Health Statistics (NCHS) at the Centers for Disease Control and Prevention (CDC) in the United States. DNA methylation array and epigenetic biomarker data were obtained from blood samples of participants. DNA was extracted from the samples and DNA methylation measurements were performed using the Illumina MethylationEPIC BeadChip. The methylation data were processed, preprocessed, and normalized to generate the epigenetic biomarker data (Table 1) (NHANES website). Sex, age, race, education level, marital status, household income, and poverty ratio were obtained from participant questionnaires and physical measurement datasets. Questionnaire data were collected using standardized questionnaires, and physical measurements were conducted at specially designed and equipped mobile centers.

**Table 1:** Biomarkers of aging.

Variable	Role	Source
HorvathAge (PMID: 24138928)	Estimated chronological age	51 tissues
HannumAge (PMID: 23177740)	Estimated chronological age	Whole blood
SkinBloodAge (PMID: 30048243)	Estimated chronological age	Skin and blood derived tissues
PhenoAge (PMID: 29676998)	Estimated chronological age	Whole blood
LinAge (PMID: 26928272)	Estimated chronological age	Whole blood
WeidnerAge (PMID: 24490752)	Estimated chronological age	Whole blood
VidalBraloAge (PMID: 27471517)	Estimated chronological age	Whole blood
YangCell (PMID: 27716309)	Forecasted mitotic cell division	Whole blood
ZhangAge (PMID: 31443728)	Estimated chronological age	Whole blood and saliva
GrimAgeMort (PMID: 30669119)	Estimated mortality	Whole blood
GrimAge2Mort (PMID: 36516495)	Estimated mortality	Whole blood
DunedinPoAm (PMID: 32367804)	predicted pace of aging	Whole blood
HorvathTelo (PMID: 31422385)	Estimated telomere length	Leukocytes
ADMMort (PMID: 30669119)	Predicted adrenomedullin	Whole blood
B2MMort (PMID: 30669119)	Predicted beta-2 microglobulin	Whole blood
CystatinCMort1 (PMID: 30669119)	predicted cystatin C	Whole blood
GDF15Mort (PMID: 30669119)	Predicted Beta-2 growth differentiation factor 15 u	Whole blood
LeptinMort (PMID: 30669119)	Predicted leptin	Whole blood
logA1CMort (PMID: 36516495)	Expected hemoglobin A1c	Whole blood
CRPMort (PMID: 36516495)	Predicted high sensitivity C-reactive protein	Whole blood
PACKYRSMort (PMID: 30669119)	Predicted pack years of smoking	Whole blood
PAI1Mort (PMID: 30669119)	Predicted plasminogen activation inhibitor	Whole blood
TIMP1Mort (PMID: 30669119)	predicted tissue inhibitor metalloproteinase 1	Whole blood
CD8TPP (PMID: 23903776, PMID: 29843789, PMID: 26956433)	Predicted CD8+ T-cell proportion	Whole blood
CD4TPP (PMID: 23903776, PMID: 29843789, PMID: 26956433)	Predicted CD4+ T-cell proportion	Whole blood
NKcel (PMID: 23903776, PMID: 29843789, PMID: 26956433)	Predicted natural killer cell proportion	Whole blood
Bcell (PMID: 23903776, PMID: 29843789, PMID: 26956433)	Predicted B-cell proportion	Whole blood
MonoPP2 (PMID: 23903776, PMID: 29843789, PMID: 26956433)	Predicted monocyte proportion	Whole blood
NeuPP (PMID: 23903776, PMID: 29843789, PMID: 26956433)	Predicted neutrophil proportion	Whole blood

In our study, race was categorized based on the questionnaire responses into five groups: Mexican American, non-Hispanic White, non-Hispanic Black, other Hispanic, and other races, with values assigned from 1 to 5. Education level was classified as high school or higher vs. less than high school. Marital status was categorized as married, cohabiting, or unmarried/not living with a partner. Household income and poverty ratio were divided into ≥300 and <300%, and BMI was categorized as ≥24 and <24.

### 2.2 Statistical analysis

Descriptive statistical analyses were performed using R software (version 4.4.1) (Tables 2, 3). For normally distributed continuous variables, means and standard deviations were reported, while for non-normally distributed continuous variables, medians and interquartile ranges were reported. Categorical variables were presented as percentages. Continuous variables were analyzed using t-tests or Mann–Whitney U tests, while categorical variables were analyzed using chi-square tests or Fisher’s exact tests.

**Table 2:** Cancer baseline table.

Variable	Non-tumor patients	Tumor patients	*p* Overall
	*N* = 1,083	*N* = 174	
Sex:			0.627
Female	523 (48.3%)	80 (46.0%)	
Male	560 (51.7%)	94 (54.0%)	
Age	63.8 (9.69)	69.1 (10.00)	<0.001
Race:			<0.001
Mexican	272 (25.1%)	23 (13.2%)	
Non-Hispanic whites	63 (5.8%)	6 (3.4%)	
Non-Hispanic blacks	459 (42.4%)	119 (68.4%)	
Other Hispanics	249 (23.0%)	25 (14.4%)	
Other Ethnic Groups	40 (3.7%)	1 (0.6%)	
Edu:			0.039
High school and above	857 (79.1%)	150 (86.2%)	
Below high school	226 (20.9%)	24 (13.8%)	
Marry:			1.000
Get married	734 (67.8%)	118 (67.8%)	
Unmarried	349 (32.2%)	56 (32.2%)	
Hypertension:			0.053
Didn’t happened	587 (54.2%)	80 (46.0%)	
Exist	496 (45.8%)	94 (54.0%)	
Diabetes:			0.278
Didn’t happened	894 (82.5%)	150 (86.2%)	
Exist	189 (17.5%)	24 (13.8%)	
HorvathAge	65.2 (8.84)	70.3 (9.81)	<0.001
HannumAge	64.8 (9.31)	70.2 (9.80)	<0.001
SkinBloodAge	62.4 (9.27)	67.4 (9.74)	<0.001
PhenoAge	53.3 (10.2)	58.9 (11.0)	<0.001
ZhangAge	65.9 (3.62)	68.0 (3.82)	<0.001
LinAge	55.3 (11.5)	61.7 (13.3)	<0.001
WeidnerAge	53.5 (10.2)	56.9 (11.8)	<0.001
VidalBraloAge	59.2 (6.27)	63.0 (7.58)	<0.001
HorvathAge acceleration	1.40 (5.32)	1.24 (6.14)	0.755
HannumAge acceleration	0.98 (5.14)	1.11 (5.64)	0.779
SkinBloodAge acceleration	−1.40 (4.12)	−1.64 (4.90)	0.526
PhenoAge acceleration	−10.45 (6.33)	−10.18 (6.90)	0.626
ZhangAge acceleration	2.15 (6.47)	−1.06 (6.82)	<0.001
LinAge acceleration	−8.43 (6.66)	−7.42 (8.51)	0.134
WeidnerAge acceleration	−10.32 (8.80)	−12.13 (10.1)	0.027
VidalBraloAge acceleration	−4.56 (7.32)	−6.08 (8.04)	0.020
GDF15Mort	928 (154)	1,010 (171)	<0.001
B2MMort	1,712,009 (168,743)	1,780,582 (166,397)	<0.001
CystatinCMort	606,425 (38,641)	625,317 (41,041)	<0.001
TIMP1Mort	35,106 (1,555)	35,939 (1,663)	<0.001
ADMMort	347 (27.5)	357 (26.8)	<0.001
PAI1Mort	17,262 (2,749)	17,685 (2,632)	0.052
LeptinMort	9,291 (4,075)	9,198 (3,992)	0.776
PACKYRSMort	18.0 (13.0)	20.8 (12.6)	0.007
CRPMort	0.73 (0.45)	0.75 (0.45)	0.508
logA1CMort	1.73 (0.03)	1.73 (0.03)	0.446
GrimAgeMort	64.2 (8.64)	69.0 (9.12)	<0.001
GrimAge2Mort	70.0 (8.55)	74.4 (9.04)	<0.001
HorvathTelo	6.63 (0.30)	6.47 (0.34)	<0.001
YangCell	0.06 (0.02)	0.07 (0.02)	0.312
DunedinPoAm	1.10 (0.09)	1.12 (0.09)	0.020
CD8TPP	0.09 (0.04)	0.09 (0.04)	0.514
CD4TPP	0.17 (0.06)	0.15 (0.07)	<0.001
Nkcell	0.06 (0.03)	0.06 (0.03)	0.158
Bcell	0.07 (0.03)	0.06 (0.05)	0.220
MonoPP	0.08 (0.02)	0.08 (0.02)	0.181
NeuPP	0.57 (0.11)	0.60 (0.12)	0.018
WTDN4YR	33,481 (31,803)	38,071 (28,596)	0.054

**Table 3:** Diabetic baseline table.

Variable	Non-diabetic	Diabetic	*p* Overall
	*N* = 1,810	*N* = 394	
Age	65.6 (10.1)	66.6 (8.99)	0.048
Race:			<0.001
Mexican	481 (26.6%)	135 (34.3%)	
Non-Hispanic whites	115 (6.4%)	30 (7.6%)	
Non-Hispanic blacks	800 (44.2%)	106 (26.9%)	
Other Hispanics	357 (19.7%)	107 (27.1%)	
Other Ethnic Groups	57 (3.1%)	16 (4.1%)	
Edu:			0.001
High school and above	1,358 (75.0%)	262 (66.5%)	
Below high school	452 (25.0%)	132 (33.5%)	
Marry:			0.918
Get married	1,170 (64.6%)	253 (64.2%)	
Unmarried	640 (35.4%)	141 (35.8%)	
Hypertension:			<0.001
Didn’t happened	1,018 (56.2%)	138 (35.0%)	
Exist	792 (43.8%)	256 (65.0%)	
HorvathAge	66.5 (9.23)	67.8 (8.43)	0.007
HannumAge	66.6 (9.75)	68.1 (9.05)	0.003
SkinBloodAge	64.1 (9.74)	65.4 (8.86)	0.006
PhenoAge	55.2 (10.8)	56.8 (9.86)	0.004
ZhangAge	66.6 (3.82)	67.1 (3.45)	0.029
LinAge	57.0 (12.5)	58.7 (11.2)	0.010
WeidnerAge	54.2 (10.9)	54.9 (9.46)	0.197
VidalBraloAge	60.2 (6.89)	60.5 (6.44)	0.326
HorvathAge acceleration	0.85 (5.56)	1.12 (6.02)	0.416
HannumAge acceleration	0.97 (5.50)	1.49 (5.29)	0.077
SkinBloodAge acceleration	−1.56 (4.51)	−1.20 (4.67)	0.161
PhenoAge acceleration	−10.45 (6.67)	−9.85 (6.85)	0.118
ZhangAge acceleration	1.00 (6.74)	0.41 (6.14)	0.094
LinAge acceleration	−8.58 (7.57)	−7.96 (7.07)	0.118
WeidnerAge acceleration	−11.44 (9.56)	−11.75 (9.15)	0.544
VidalBraloAge acceleration	−5.45 (7.58)	−6.11 (7.47)	0.116
GDF15Mort	962 (167)	983 (149)	0.014
B2MMort	1,743,827 (175,105)	1,769,706 (171,428)	0.007
CystatinCMort	614,217 (40,737)	620,710 (37,297)	0.002
TIMP1Mort	35,388 (1,613)	35,655 (1,421)	0.001
ADMMort	350 (27.0)	353 (25.6)	0.018
PAI1Mort	17,179 (2,752)	18,550 (2,703)	<0.001
LeptinMort	9,330 (4,053)	9,958 (4,299)	0.008
PACKYRSMort	18.8 (13.4)	19.4 (12.1)	0.369
CRPMort	0.75 (0.46)	0.93 (0.44)	<0.001
logA1CMort	1.72 (0.03)	1.75 (0.03)	<0.001
GrimAgeMort	65.8 (8.93)	67.3 (7.94)	<0.001
GrimAge2Mort	71.5 (8.82)	73.6 (7.79)	<0.001
HorvathTelo	6.58 (0.31)	6.56 (0.31)	0.220
YangCell	0.06 (0.02)	0.07 (0.02)	0.048
DunedinPoAm	1.11 (0.09)	1.12 (0.09)	0.125
CD8TPP	0.09 (0.04)	0.10 (0.05)	0.237
CD4TPP	0.17 (0.06)	0.16 (0.07)	0.503
Nkcell	0.06 (0.03)	0.06 (0.03)	0.616
Bcell	0.07 (0.03)	0.07 (0.03)	0.877
MonoPP	0.08 (0.02)	0.08 (0.02)	0.938
NeuPP	0.59 (0.11)	0.59 (0.11)	0.718
WTDN4YR	32,234 (30,634)	21,961 (25,770)	<0.001

### 2.3 Machine learning modeling

The study population was randomly divided into training and validation sets at an 80:20 ratio, followed by standardization preprocessing. Nine machine learning algorithms, including AdaBoost, GBM, KNN, LightGBM, MLP, RF, SVM, XGBoost, and Logistic Regression (see Table 4 for details), were used to develop predictive models.

**Table 4:** Comparison of differences among 9 machine learning models.

Name	Features	Advantages	Disadvantages	Applicable Scenarios
AdaBoost (Adaptive boosting)	Gradually improve model performance by combining multiple weak classifiers (typically decision trees), with a particular emphasis on samples that were previously misclassified.	Improve classification accuracy; simple and easy to implement; applicable to binary classification problems.	Sensitive to outliers and noise; difficult to handle multi-class problems.	Binary classification tasks; scenarios where the performance of simple models needs to be improved.
GBM (Gradient boosting machine)	Optimize the loss function by gradually adding decision trees and use gradient descent methods to minimize error.	Powerful predictive ability; high flexibility; capable of optimizing various loss functions.	Long training time; prone to overfitting; complex parameter tuning.	Regression and classification tasks; prediction problems on complex datasets.
KNN (K-nearest neighbors)	Based on distance metrics; make predictions by finding the nearest K neighbors; applicable to classification and regression.	Simple and intuitive; no training process required; suitable for small datasets.	High computational cost; poor performance on high-dimensional data; sensitive to noise.	Classification and regression tasks; small-scale datasets; scenarios requiring simple models.
LightGBM (Light gradient boosting machine)	An efficient gradient boosting framework developed by Microsoft, utilizing histogram-based decision tree algorithms, supporting categorical features, offering fast training speed, and low memory consumption.	Fast training speed; high memory efficiency; supports large-scale data; efficient handling of categorical features.	Complex parameter tuning; sensitive to outliers; not suitable for small datasets.	Large-scale datasets; tasks requiring fast training and prediction; scenarios with many categorical features.
MLP (Multi-layer perceptron)	A multilayer perceptron consists of multiple hidden layers, uses nonlinear activation functions, and is capable of learning complex patterns and relationships.	Able to capture complex nonlinear relationships; suitable for various tasks (classification, regression).	Long training time; requires large amounts of data; difficult to interpret; prone to overfitting.	Large-scale and complex datasets; tasks requiring handling of nonlinear relationships.
RF (Random forest)	By constructing multiple decision trees and combining their prediction results, randomly selecting features and samples to generate diverse tree structures.	Strong resistance to overfitting; suitable for high-dimensional data; capable of handling missing values; easy to parallelize.	Large model size; slow prediction speed; difficult to interpret.	Classification and regression tasks; high-dimensional data; scenarios requiring robust models.
SVM (Support vector machine)	Finds a hyperplane that maximizes the classification margin; applicable to high-dimensional and small-sample data; handles nonlinear boundaries through kernel functions.	Performs well in high-dimensional spaces; suitable for small-sample data; handles nonlinear problems through kernel functions.	Long training time; not friendly to large-scale datasets; complex parameter tuning; sensitive to noise.	High-dimensional data; small-scale datasets; tasks requiring precise classification boundaries.
XGBoost (Extreme gradient boosting)	An optimized gradient boosting framework with efficient implementation and strong performance, incorporating parallel processing, regularization, and pruning techniques.	High performance; strong scalability; supports parallel computing; prevents overfitting; widely used in competitions and real-world problems.	Parameter tuning is complex; training time is relatively long; and there are high requirements for data preprocessing.	Regression and classification tasks of various types; scenarios requiring high prediction accuracy and model performance; large-scale datasets.
Logistic regression	Transforms the linearly combined input features into probability values using a logistic function (e.g., the Sigmoid function), suitable for binary and multiclass classification problems.	Simple and easy to understand; computationally efficient; strong model interpretability; suitable for linearly separable data.	Only applicable to linear relationships; sensitive to multicollinearity; not suitable for handling complex nonlinear relationships.	Binary and multiclass classification tasks; scenarios where model interpretability is required; linearly separable datasets.

**Figure 1:**
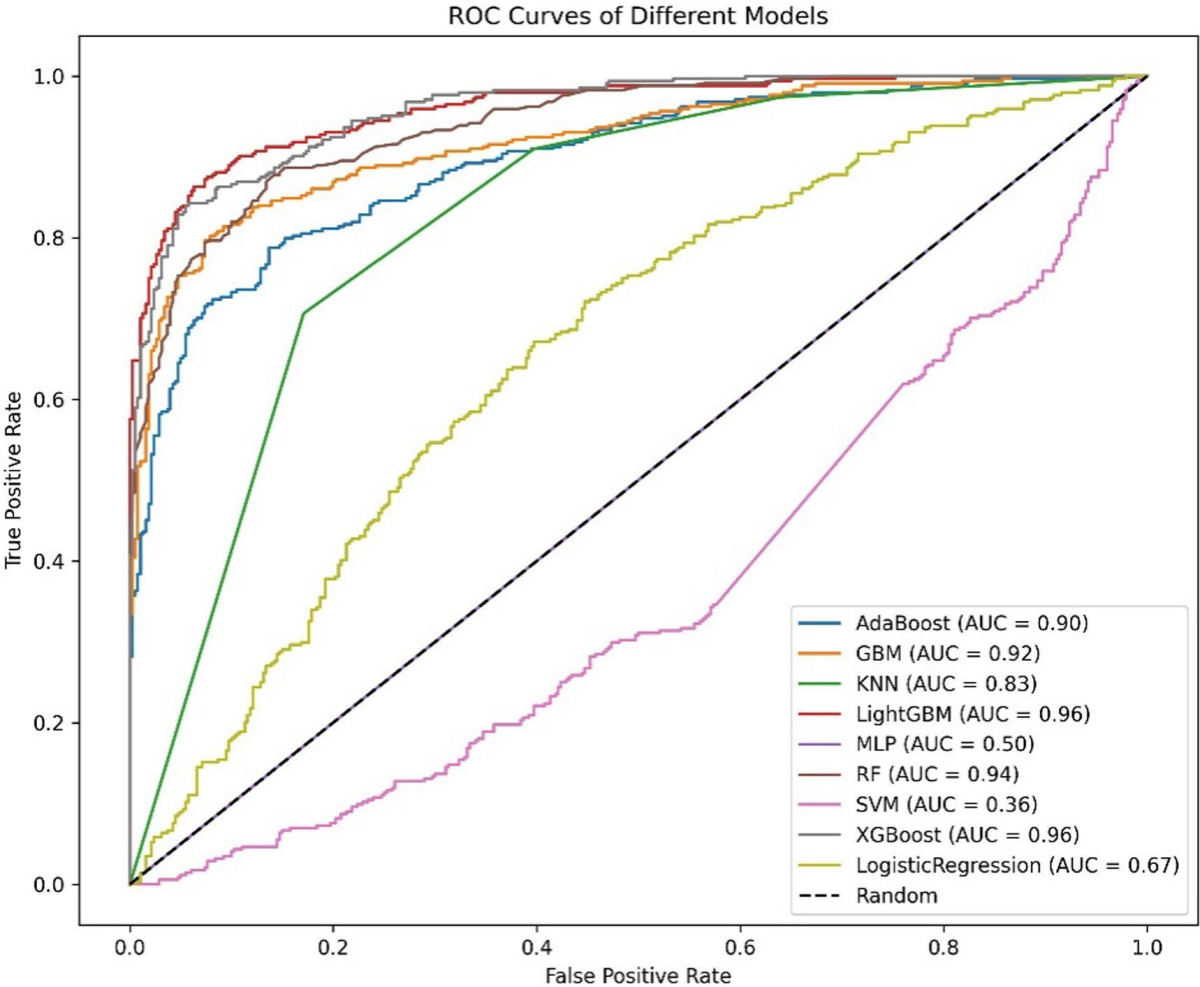
ROC curve of the diabetes model.

**Figure 2:**
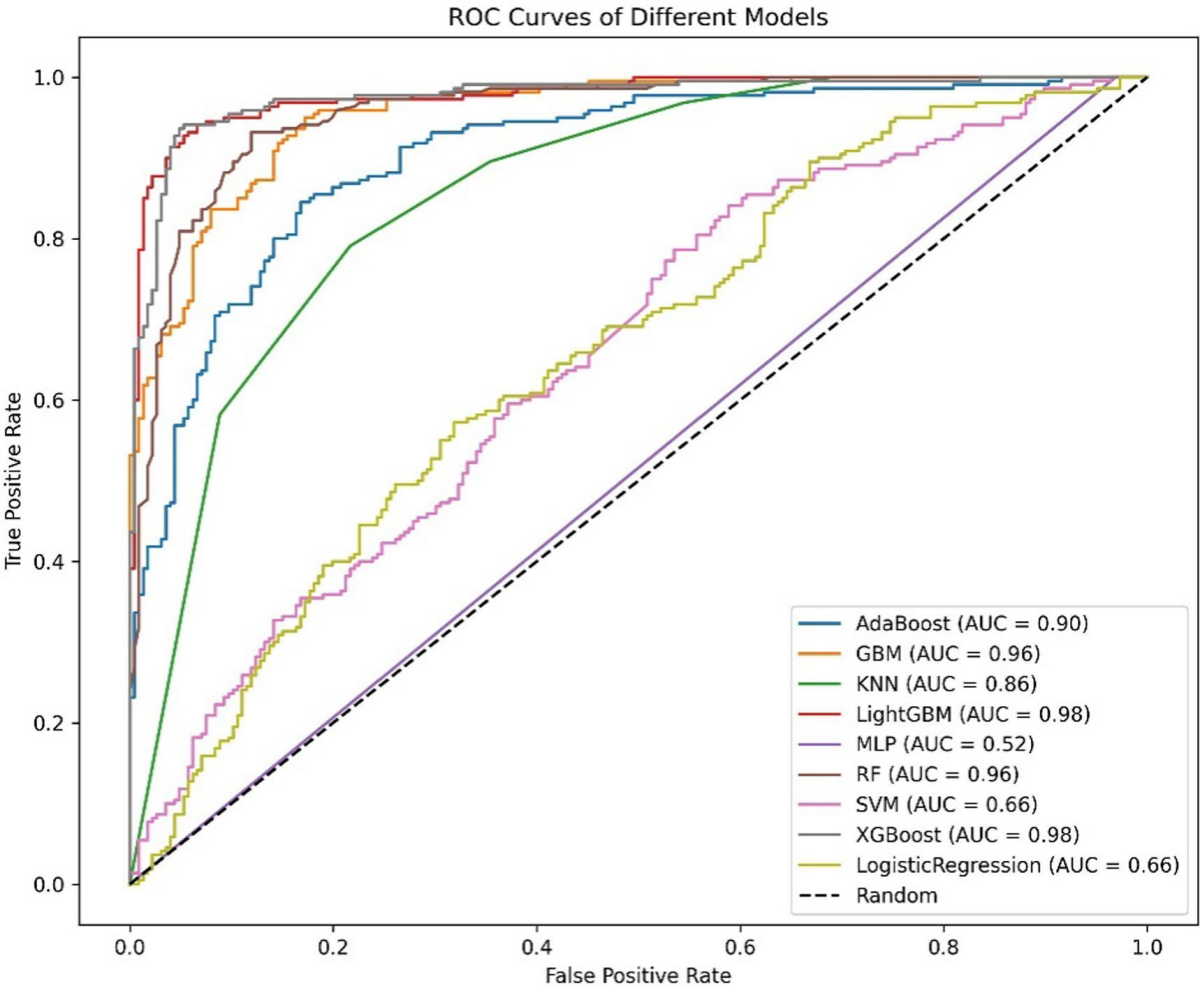
ROC curves of the cancer model.

**Figure 3:**
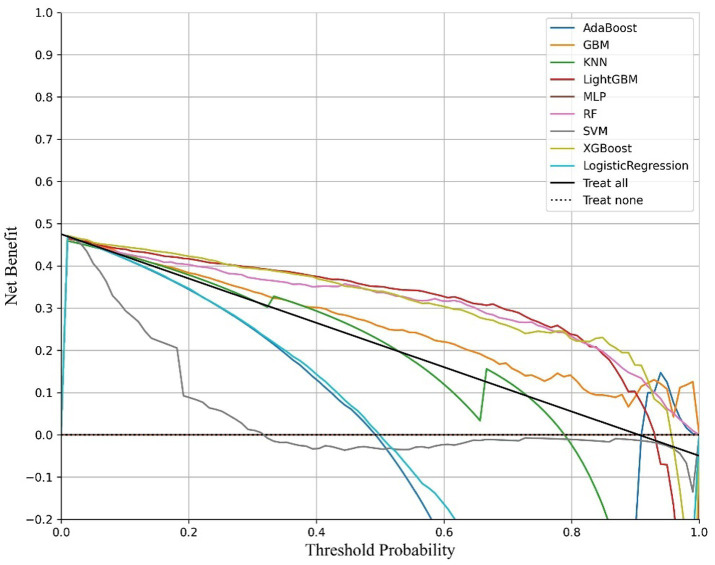
DCA curves of the diabetic model.

**Figure 4:**
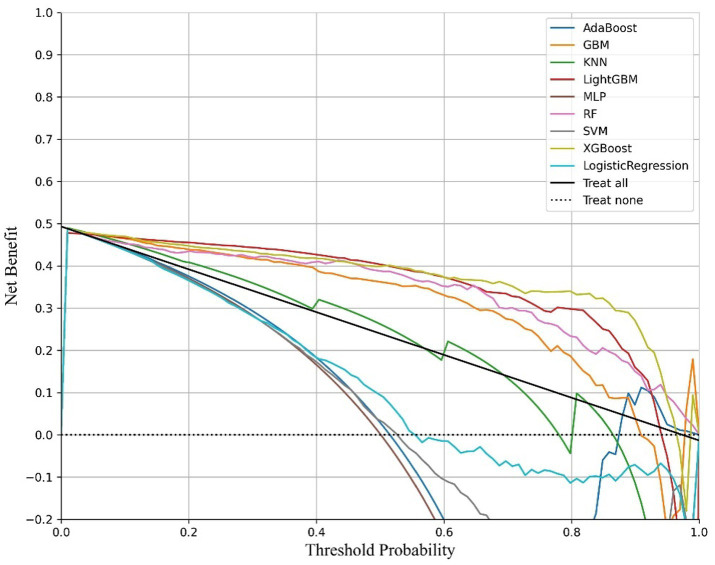
DCA curves of the cancer model.

Oversampling was performed using SMOTE to balance the positive and negative samples, generating more positive samples to alleviate the class imbalance issue. The dataset was then divided into training and test sets. CV was set to 5, indicating 5-fold cross-validation, which was used to assess the average performance of the model under different parameter combinations. The training dataset was randomly divided into five mutually exclusive subsets (folds), with one subset used as the validation set in each iteration, and the remaining four subsets combined for training. This process allowed for 5 training and validation cycles, resulting in five validation outcomes. Grid search (GridSearchCV) was used in conjunction with cross-validation to exhaustively search the parameter grid and return the optimal parameter combination for the machine learning model, thereby achieving the best performing model. Metrics such as Positive Predictive Value, Negative Predictive Value, Sensitivity, Specificity, Matthews Correlation Coefficient, AUC, Kappa, Brier Score, accuracy, and F1 Score were used to evaluate model performance (Tables 5, 6). The Positive Predictive Value (PPV) reflects the accuracy of the model’s positive predictions, with higher values indicating fewer false positives. The F1 score ranges from 0 to 1, with higher values indicating a better balance between precision and recall, making it suitable for imbalanced class problems. The above metrics, combined with ROC curves (Figures 1, 2) and DCA curves (Figures 3, 4), were used to assess the stability and clinical decision-making ability of the model (23).

**Table 5:** Diabetes prediction model performance evaluation.

Model	PPV	NPV	Sensitivity	Specificity	MCC	AUC	Kappa	Brier Score	F1	Accuracy	Precision	Recall
AdaBoost	0.66	0.88	0.91	0.58	0.52	0.9	0.49	0.25	0.77	0.74	0.66	0.91
GBM	0.71	0.89	0.91	0.67	0.59	0.92	0.57	0.15	0.8	0.78	0.71	0.91
GradientBoosting	0.72	0.89	0.91	0.67	0.59	0.92	0.57	0.15	0.8	0.78	0.72	0.91
KNN	0.67	0.88	0.91	0.6	0.53	0.83	0.5	0.19	0.77	0.75	0.67	0.91
LightGBM	0.84	0.92	0.92	0.84	0.76	0.96	0.75	0.09	0.88	0.88	0.84	0.92
MLP	0.0	0.52	0.0	1.0	0.0	0.5	0.0	0.48	0.0	0.52	1.0	0.0
RF	0.86	0.86	0.85	0.87	0.72	0.94	0.72	0.11	0.85	0.86	0.86	0.85
SVM	0.48	0.0	1.0	0.0	0.0	0.36	0.0	0.26	0.64	0.48	0.48	1.0
XGBoost	0.82	0.91	0.91	0.82	0.73	0.96	0.73	0.1	0.87	0.86	0.82	0.91
LogisticRegression	0.48	0.76	0.98	0.05	0.09	0.67	0.03	0.34	0.65	0.49	0.48	0.98

**Table 6:** Cancer prediction model performance evaluation.

Model	PPV	NPV	Sensitivity	Specificity	MCC	AUC	Kappa	Brier Score	F1	Accuracy	Precision	Recall
AdaBoost	0.71	0.92	0.94	0.63	0.6	0.9	0.57	0.24	0.81	0.78	0.71	0.94
GBM	0.81	0.95	0.96	0.78	0.75	0.96	0.74	0.1	0.88	0.87	0.81	0.96
GradientBoosting	0.81	0.95	0.96	0.78	0.75	0.96	0.74	0.1	0.88	0.87	0.81	0.96
KNN	0.71	0.86	0.9	0.65	0.56	0.86	0.54	0.17	0.79	0.77	0.71	0.9
LightGBM	0.87	0.96	0.96	0.86	0.83	0.98	0.82	0.07	0.91	0.91	0.87	0.96
MLP	0.5	1.0	1.0	0.03	0.12	0.52	0.03	0.49	0.67	0.51	0.5	1.0
RF	0.89	0.91	0.9	0.89	0.79	0.96	0.79	0.09	0.9	0.9	0.89	0.9
SVM	0.49	0.0	1.0	0.0	0.0	0.66	0.0	0.23	0.66	0.49	0.49	1.0
XGBoost	0.86	0.97	0.97	0.84	0.82	0.98	0.81	0.07	0.91	0.91	0.86	0.97
LogisticRegression	0.49	0.0	1.0	0.0	0.0	0.66	0.0	0.34	0.66	0.49	0.49	1.0

**Figure 5:**
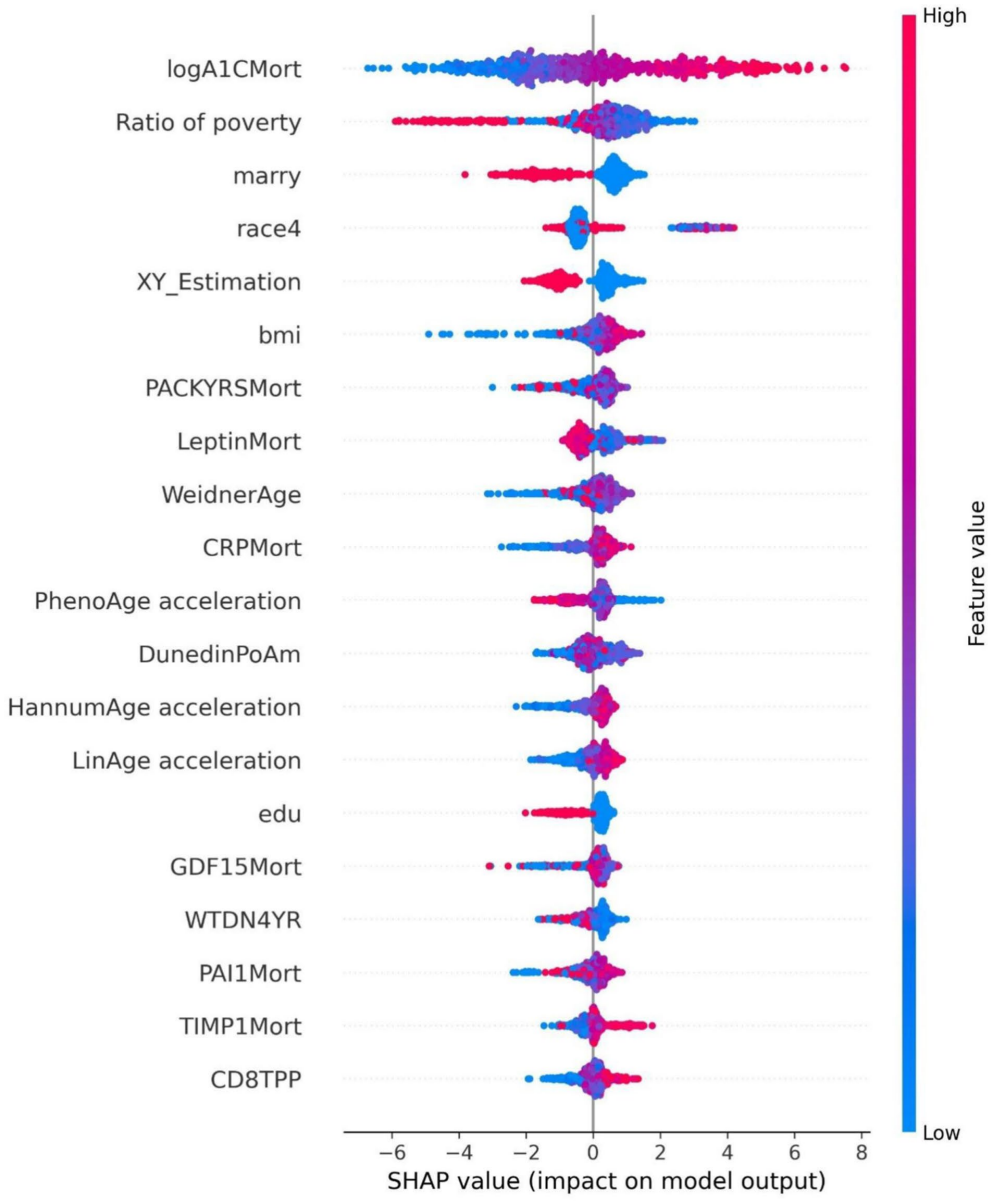
Summary plot of SHAP values for the diabetes model.

**Figure 6:**
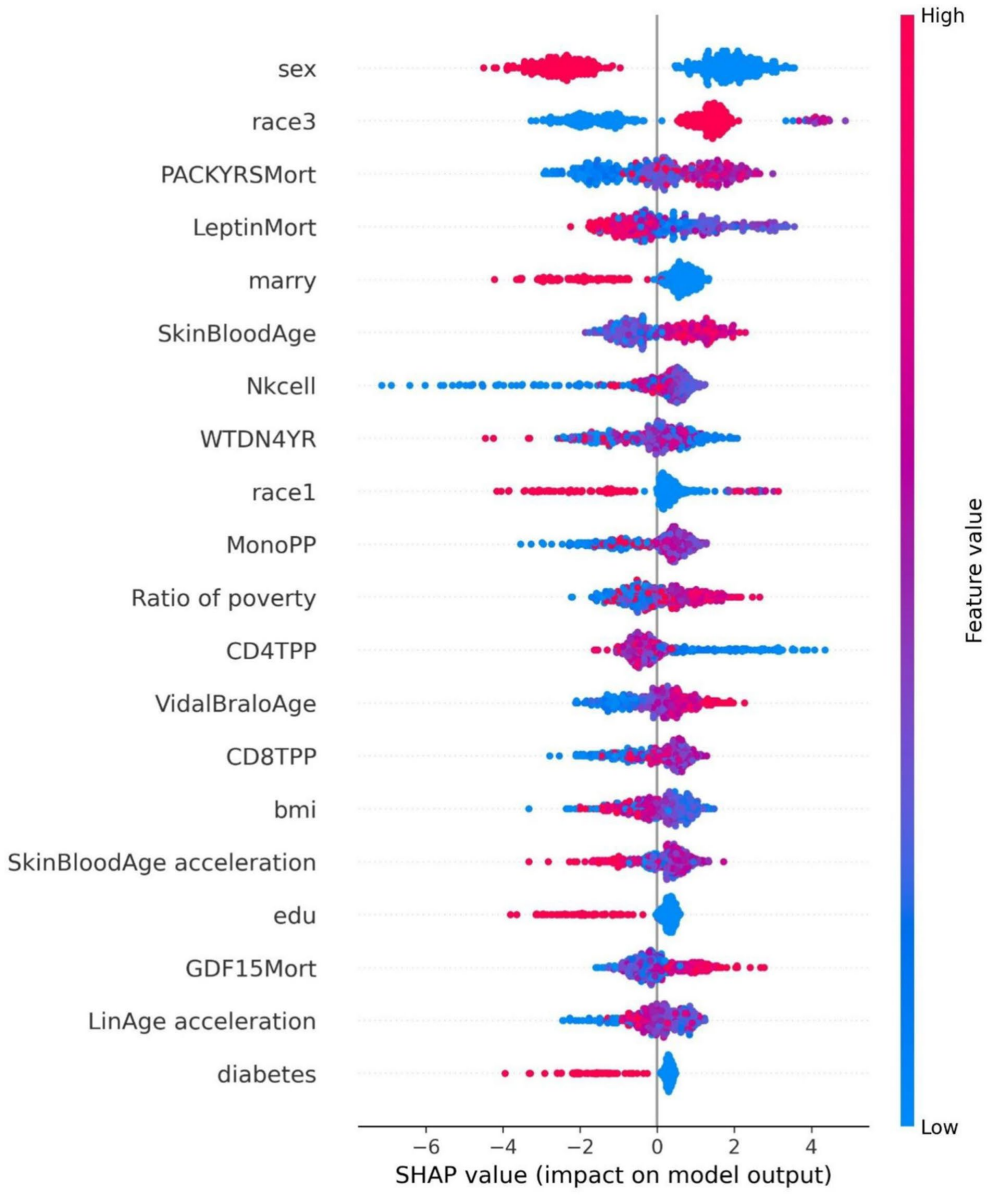
Summary plot of SHAP values for the cancer model.

**Figure 7:**
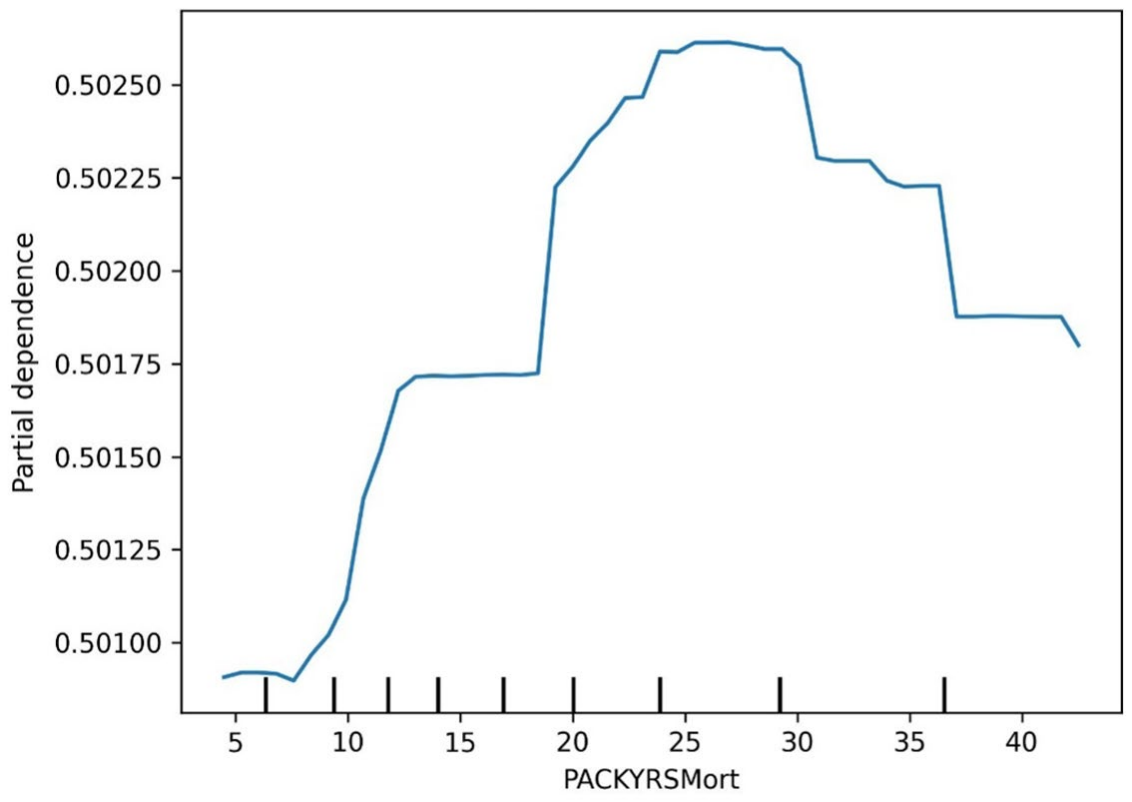
Cancer PACKYSMort partial dependence plot.

**Figure 8:**
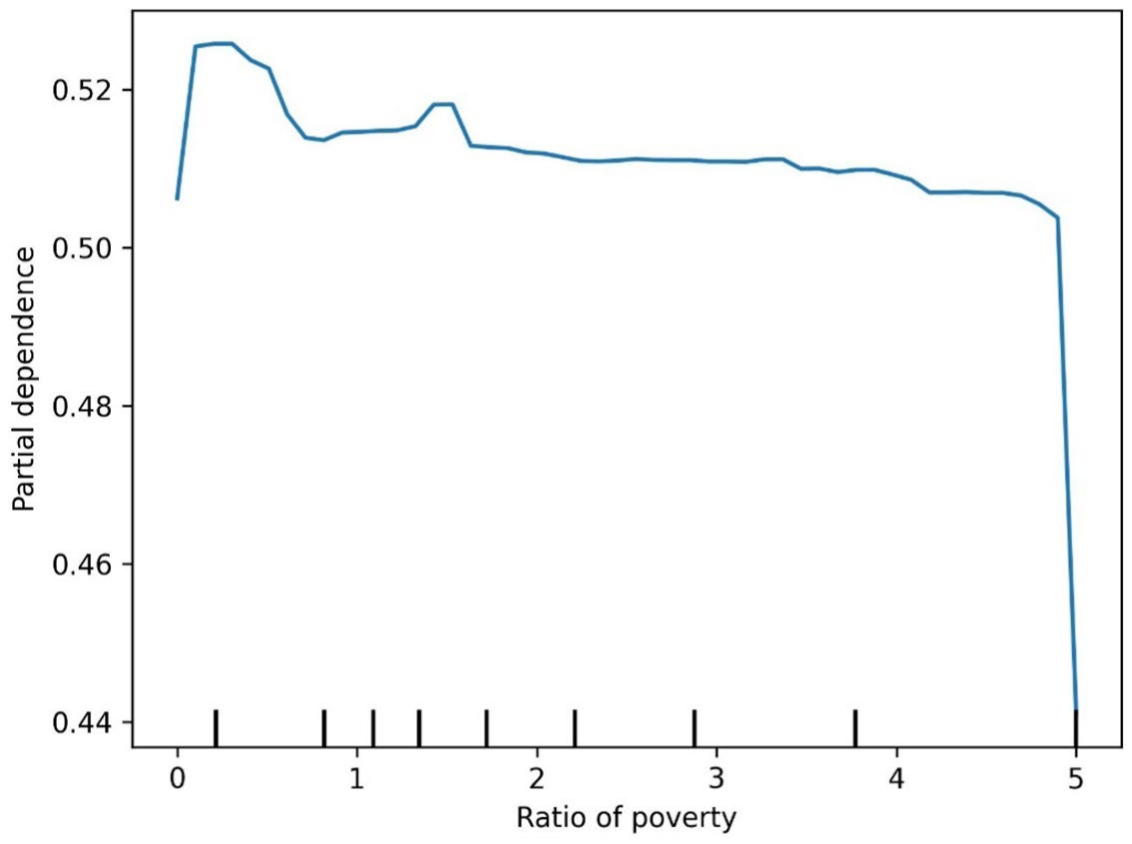
Diabetes ratio of poverty partial dependence plot.

**Figure 9:**
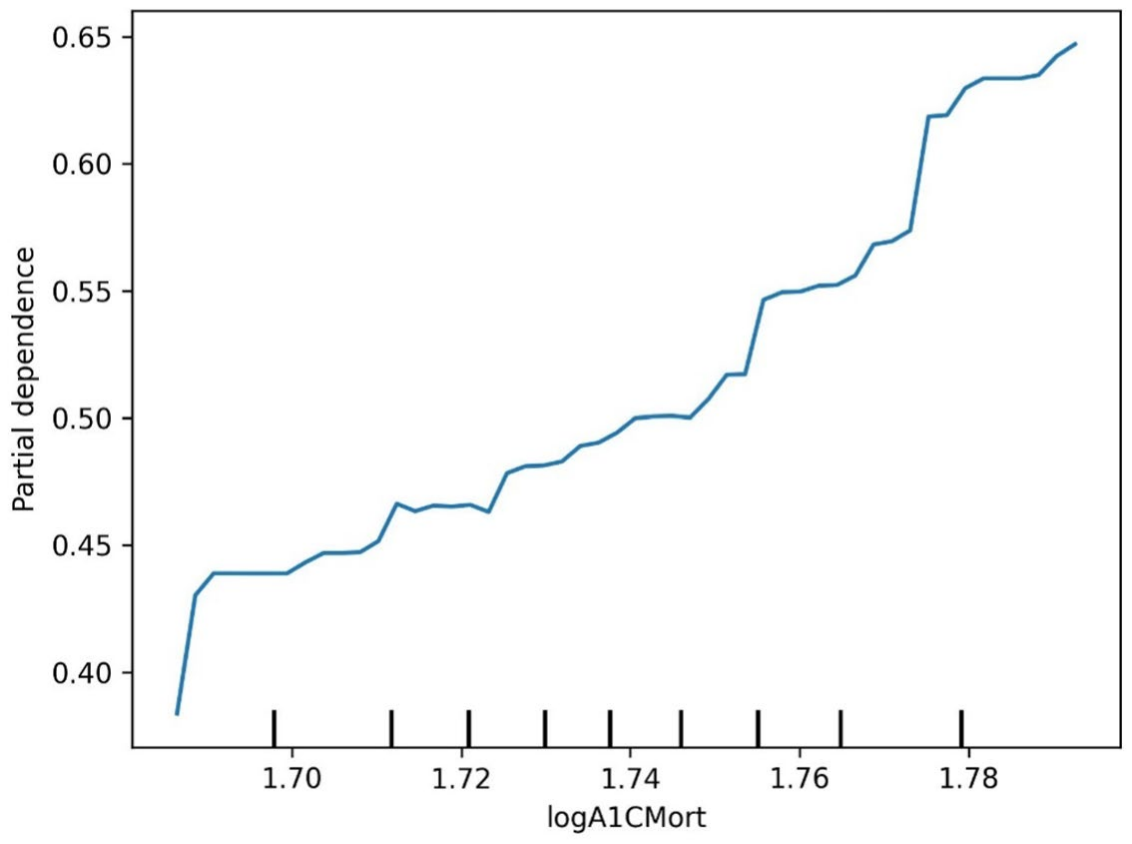
Diabetes logA1CMort partial dependence plot.

The ROC curve (Receiver Operating Characteristic Curve) measures the model’s discriminatory ability at various thresholds by plotting the trade-off between the True Positive Rate (TPR) and False Positive Rate (FPR). It is insensitive to the proportion of positive and negative samples and is ideal for evaluating binary classification models comprehensively. The DCA curve is typically used to assess the “net benefit” of a model in practical clinical applications, taking into account the risk–benefit balance at different thresholds. Net benefit is a metric that combines the risks of false positives and false negatives. A high net benefit indicates that the model effectively identifies the disease at that threshold, with a greater contribution compared to the cost of incorrect predictions. The threshold refers to the probability cutoff used by the decision model, which determines whether a sample is classified as positive. A higher threshold reduces false positives but may miss some true positives (i.e., increasing false negatives). We need to select the model that provides higher net benefit across a broader range of thresholds. The best-performing model was selected, and SHAP values were computed and plotted in summary graphs (Figures 5, 6) to report the contribution of each feature. Partial dependence plots (Figures 7–9) were used to visualize the impact of changes in aging-related genetic biomarkers on the outcomes.

## 3 Results

### 3.1 Baseline table

Table 3 the diabetes model included a total of 2,205 participants, with 394 diabetic patients, the majority of whom were of other Hispanic descent. The average age of diabetic patients was 66.6 years (SD 8.99), significantly higher than that of non-diabetic patients, which was 65.6 years (SD 10.1), *p* = 0.048, indicating that age is an important risk factor for tumor occurrence. There was also a significant difference in racial distribution between the two groups (*p* < 0.001). The proportion of Mexican Americans (34.3%) and other Hispanics (27.1%) was significantly higher in diabetic patients compared to non-diabetic patients (26.6 and 19.7%, respectively), while the proportion of non-Hispanic Blacks was significantly lower in diabetic patients (26.9%) compared to non-diabetic patients (44.2%), indicating a significant racial difference in tumor incidence. The proportion of diabetic patients with a high school education or higher was significantly lower than that of non-diabetic patients (66.5% vs. 75.0%, *p* = 0.001). In addition, the prevalence of hypertension was significantly higher in diabetic patients compared to non-diabetic patients (65.0% vs. 43.8%, *p* < 0.001). There was no significant difference in marital status distribution between the two groups (*p* = 0.918). Age-related indicators: All biological age markers (such as HorvathAge, HannumAge, PhenoAge, etc.) in the diabetic group were significantly higher than those in the non-diabetic group (*p* < 0.05). Indicators such as HorvathAge acceleration, HannumAge acceleration, SkinBloodAge acceleration, PhenoAge acceleration, ZhangAge acceleration, and LinAge acceleration showed significant differences in diabetic patients (*p* < 0.05), While some age acceleration indicators, such as WeidnerAge acceleration and VidalBraloAge acceleration, showed no significant differences in diabetic patients (*p* > 0.05). Indicators such as GDF15Mort, B2MMort, CystatinCMort, TIMP1Mort, ADMMort, PAI1Mort, LeptinMort, CRPMort, logA1CMort, GrimAgeMort, GrimAge2Mort, and YangCell were significantly higher in diabetic patients than in non-diabetic patients (*p* < 0.05). Indicators such as PACKYRSMort, HorvathTelo, DunedinPoAm, CD8TPP, CD4TPP, Nkcell, Bcell, MonoPP, and NeuPP showed no significant differences between the two groups (*p* > 0.05).

Table 2 the cancer model included a total of 1,291 participants, with 177 cancer patients, primarily distributed among non-Hispanic Black individuals. The average age of cancer patients (69.1 years, SD 10.00) was significantly higher than that of non-cancer patients (63.8 years, SD 9.69), *p* < 0.001. The proportion of cancer patients with a high school education or higher (86.2%) was significantly higher than that of non-cancer patients (79.1%), *p* = 0.039. There was a significant difference in racial distribution between the two groups (*p* < 0.001). Among non-cancer patients, the proportion of non-Hispanic Black individuals was relatively low (42.4%), while this proportion significantly increased to 68.4% in cancer patients. Additionally, the proportion of Mexican Americans, other Hispanics, and other ethnic groups was significantly lower among cancer patients. There was no significant difference between the two groups in terms of marital status (Marry), prevalence of hypertension (Hypertension), and incidence of diabetes (Diabetes). All age clock indicators (such as HorvathAge, HannumAge, SkinBloodAge, PhenoAge, ZhangAge, LinAge, WeidnerAge, VidalBraloAge, etc.) were significantly higher in cancer patients than in non-cancer patients (*p* < 0.001). ZhangAge acceleration: Cancer patients had significantly lower values than non-cancer patients (*p* < 0.001). WeidnerAge acceleration (*p* = 0.027) and VidalBraloAge acceleration (*p* = 0.020): The acceleration markers in cancer patients were significantly different from those in non-cancer patients. Other age acceleration markers (such as HorvathAge acceleration, HannumAge acceleration, etc.) showed no significant differences (*p* > 0.05). Indicators such as GDF15Mort, B2MMort, CystatinCMort, TIMP1Mort, ADMMort, PACKYRSMort, GrimAgeMort, GrimAge2Mort, HorvathTelo, DunedinPoAm, CD4TPP, and NeuPP were significantly higher in cancer patients than in non-cancer patients (*p* < 0.05). Indicators such as LeptinMort, CRPMort, logA1CMort, YangCell, CD8TPP, Nkcell, Bcell, and MonoPP showed no significant differences between the two groups (*p* > 0.05), suggesting that these markers may not have a direct association with tumor incidence in this study, or their mechanisms of action are complex and require further exploration with larger sample sizes or multivariate analyses.

### 3.2 Epigenetic age clocks

Correlation analysis shows that the correlation coefficients between eight epigenetic ages and chronological age are all greater than 0.5 (Table 7), indicating that they are all strongly positively correlated with age, thus confirming the reliability of these clocks.

**Table 7:** Correlation analysis of chronological age and epigenetic age.

Variable	Correlation coefficient
HorvathAge	0.847
HannumAge	0.861
SkinBloodAge	0.910
PhenoAge	0.804
ZhangAge	0.930
LinAge	0.811
WeidnerAge	0.571
VidalBraloAge	0.662

**Figure 10:**
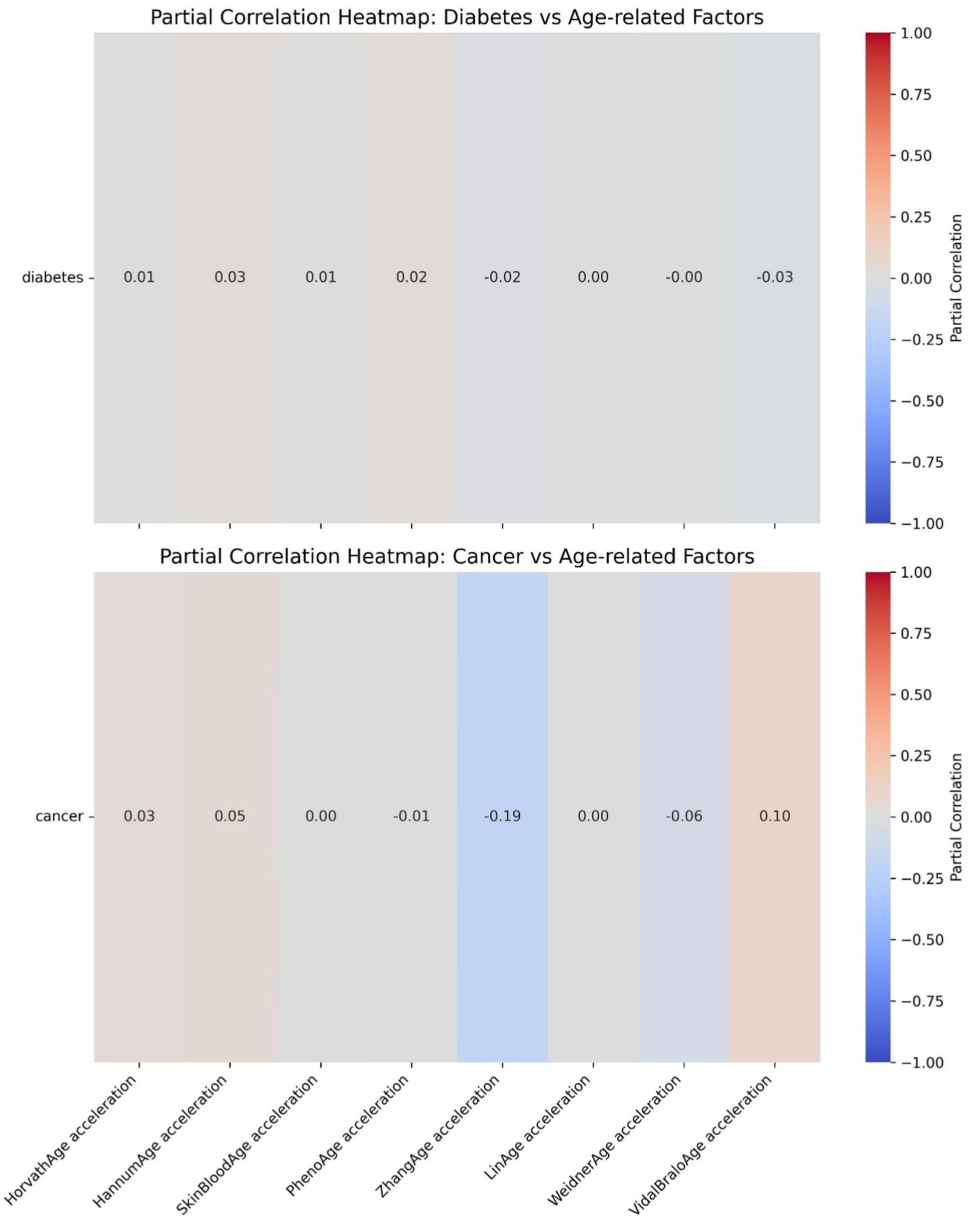
Epigenetic age acceleration correlation heatmaps.

**Figure 11:**
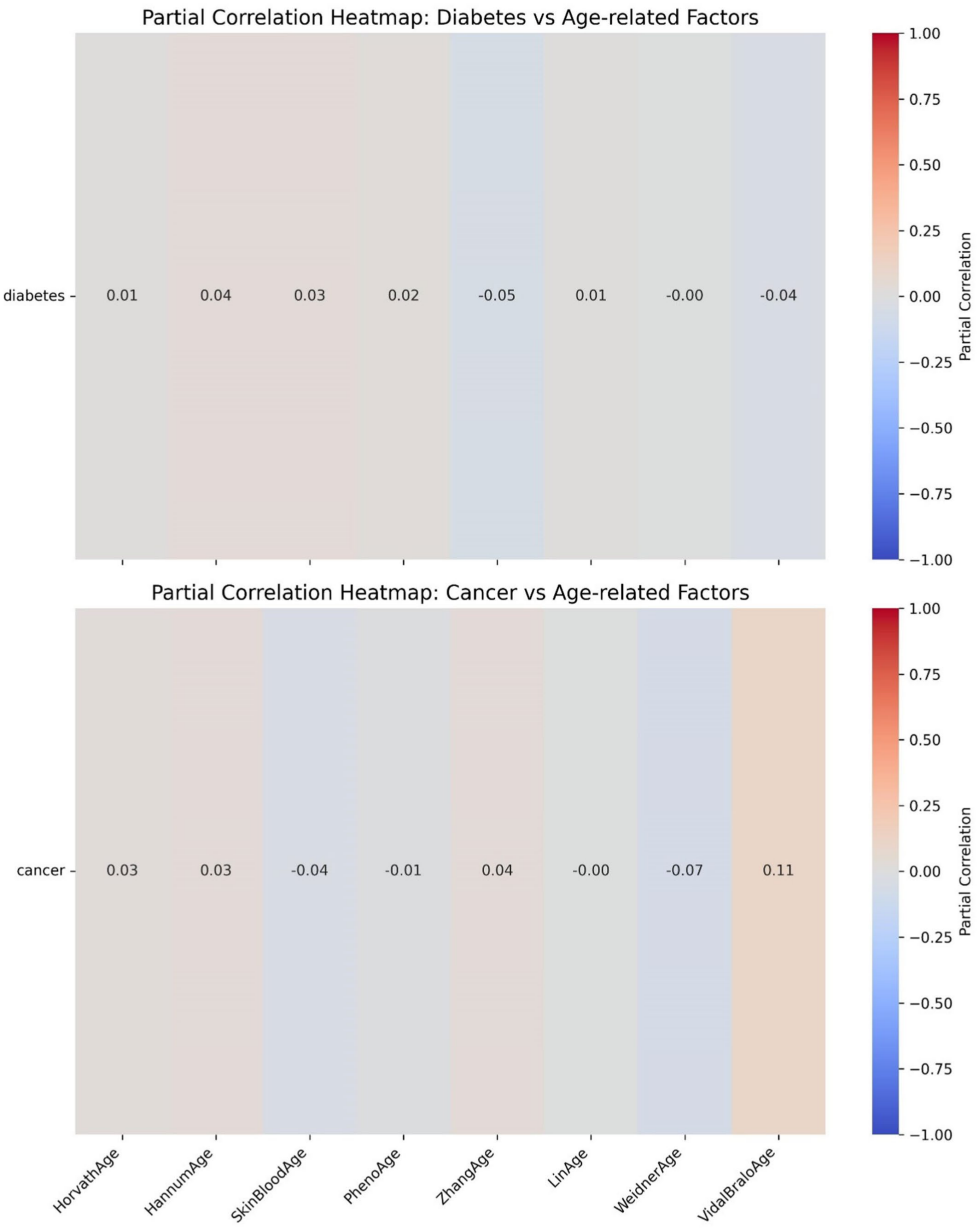
Epigenetic age acceleration correlation heatmaps.

Further heatmap analysis was performed to examine the correlation between epigenetic age acceleration (epigenetic age – chronological age) and diabetes, as well as cancer (Figures 10, 11). These two heatmaps display the partial correlation coefficients between diseases (diabetes and cancer) and epigenetic age clock acceleration, illustrating the independent correlations between each disease and different age acceleration factors. Partial correlation refers to the correlation coefficient for each number, representing the partial correlation between a specific disease (diabetes or cancer) and a particular age acceleration factor. Positive correlation (red): A positive coefficient indicates that the disease is positively correlated with the acceleration factor (the higher the acceleration factor, the higher the disease risk). Negative correlation (blue): A negative coefficient indicates that the disease is negatively correlated with the acceleration factor (the higher the acceleration factor, the lower the disease risk). Close to 0 (gray): No significant correlation. In the heatmap, the colors range from red (positive correlation) to blue (negative correlation), with gray indicating weak correlations.

The partial correlation coefficients are generally close to 0, indicating that there is almost no significant independent correlation between diabetes and these epigenetic age clock accelerations. The differences in the effects of each acceleration factor on diabetes are minimal and not statistically significant.

Figure 10 LinAge acceleration and ZhangAge acceleration show a weak negative correlation with cancer (−0.19), suggesting that these two acceleration factors may be associated with a reduced risk of cancer. VidalBraloAge acceleration and HannumAge acceleration show a weak positive correlation with cancer (0.10 and 0.05, respectively), indicating that these epigenetic age clock accelerations may be associated with an increased risk of cancer. Other acceleration factors (such as HorvathAge, SkinBloodAge, etc.) show a correlation close to 0 with cancer.

### 3.3 Development and performance of risk models

After calculating evaluation metrics and plotting the ROC and DCA curves, this study assesses model performance using multiple indicators, with detailed evaluation values provided in Tables 5, 6, which present the performance evaluation of the diabetes and cancer prediction models. This study plots the ROC curves for various models of diabetes and cancer. According to the definition of the ROC curve, as the threshold changes, the curve should ideally be as “convex” as possible toward the top-left corner. Regions with high TPR and low FPR indicate better classifier performance, so LightGBM is chosen as the model with the best performance. Models with higher DCA curves indicate greater net benefit at the corresponding threshold probabilities, thus demonstrating superior performance. Different models may perform differently across different threshold ranges. For example, in the low threshold range (<0.2), some models (such as Logistic Regression and LightGBM) may have higher net benefits. In the middle range (0.2–0.6), models like Gradient Boosting and RF may outperform others. At high thresholds (>0.7), the net benefit of the models may approach that of the “Treat None” strategy (no intervention). The reference significance of “Treat All” and “Treat None”: “Treat All” is an extreme strategy where all patients are treated. “Treat None” is another extreme strategy where no patient receives intervention. When the model’s curve lies between “Treat All” and “Treat None,” it indicates that the model’s prediction lacks clinical value. Selection of the optimal model: Considering the overall threshold range, the model that consistently stays above “Treat All” and “Treat None” while covering a broader threshold probability range is the optimal choice. From this DCA curve, it is observed that Gradient Boosting and LightGBM demonstrate superior net benefits across larger threshold ranges. This study evaluates model performance using various indicators, with detailed evaluation values provided in Tables 5, 6, which present the performance evaluation of the diabetes and cancer prediction models. In the diabetes prediction, LightGBM achieved an AUC of 1.00, an MCC of 0.97, and an F1 Score of 0.98 on the validation set, while GBM had an AUC of 0.99, an MCC of 0.93, and an F1 Score of 0.95 (Table 5). LightGBM is thus selected as the best model. Similarly, in cancer prediction, LightGBM achieved an AUC of 0.98, an MCC of 0.83, and an F1 Score of 0.91 (Table 6). Therefore, it can be concluded that the LightGBM model is the best comprehensive model for assessing the risk of diabetes and cancer, and is selected for use.

The SHAP value summary plot reports that the top three contributing features in the diabetes model are logA1Mort, family income-to-poverty ratio, and marital status (Figure 5). In the cancer model, the top three contributing features are gender, non-Hispanic white, and PACKYRSMort (Figure 6). Based on the above SHAP value summary plot, partial dependence plots for important features were drawn. An increase in logA1Mort is positively correlated with the risk of diabetes (Figure 9). When the economic status of the subjects is poorer (such as when the family income-to-poverty ratio is close to 0 or 1), the probability of developing diabetes is higher. When the family income-to-poverty ratio is between 1 and 4, the partial dependence values stabilize, and there is no significant fluctuation in diabetes risk. When the poverty ratio exceeds 4, the partial dependence values sharply decrease, suggesting that a higher poverty ratio (better economic status) may be associated with a lower risk of diabetes (Figure 8). On the other hand, Figure 7 shows that PACKYRSMort is non-linearly related to cancer risk. When the cumulative smoking burden is less than 10, the risk of cancer is relatively low. When the cumulative smoking burden is between 10 and 30, the risk of cancer significantly increases. When the cumulative smoking burden exceeds 30, the risk of cancer shows a declining trend.

## 4 Discussion

### 4.1 Epigenetic age clock

Epigenetic clocks rely on DNA methylation (DNAm) analysis at specific CpG sites to predict an individual’s chronological age with high accuracy (11). Additionally, epigenetic age acceleration—the discrepancy between chronological age and epigenetic age—is increasingly recognized as a marker of aging, capable of predicting premature onset of diseases and early mortality (12). When applied to large-scale human epidemiological datasets, research has shown that the gap between predicted epigenetic age and actual chronological age is linked to various age-related diseases, particularly when epigenetic age surpasses chronological age. There is growing evidence supporting the notion that accelerated epigenetic aging serves as a novel biomarker for cancer risk. A study by Plonski et al. (13) found that epigenetic age acceleration correlates with late-stage mortality in both cancer and chronic diseases. Similarly, research by Zheng et al. (24) demonstrated a statistically significant positive correlation between epigenetic age acceleration and overall mortality, highlighting its role as a key predictor of colorectal cancer survival. Levine et al. (25) further suggested that accelerated epigenetic aging could serve as a useful biomarker for assessing lung cancer susceptibility. In the present study, univariate analysis revealed that all epigenetic clocks (including HorvathAge, HannumAge, PhenoAge, and others) were significantly elevated in cancer patients compared to non-cancer controls. Heatmap analysis also identified a weak negative correlation (−0.19) between LinAge acceleration and ZhangAge acceleration with cancer risk, suggesting that these two forms of epigenetic age acceleration might be associated with a lower cancer risk. Conversely, VidalBraloAge acceleration and HannumAge acceleration showed weak positive correlations (0.10 and 0.05, respectively) with cancer, indicating that these epigenetic age accelerations may be linked to an increased cancer risk. These findings support the conclusion that epigenetic age acceleration is potentially associated with cancer risk. Consistent with previous studies (14), which have suggested that epigenetic age accelerations, such as Horvath acceleration, Hannum acceleration, and Grim acceleration, can predict cancer mortality, the results reinforce the role of epigenetic age acceleration in cancer outcomes.

In this study, various epigenetic age acceleration markers, including HorvathAge accelerations, HannumAge accelerations, SkinBloodAge accelerations, PhenoAge accelerations, ZhangAge accelerations, and LinAge accelerations, exhibited significant differences in diabetic patients (*p* < 0.05). However, there was little to no significant independent correlation between diabetes and these accelerations of epigenetic age clocks. Previous research (8) has also shown that epigenetic age is linked to the mortality risk in individuals with type 2 diabetes. Vetter et al. (26) noted that after adjusting for covariates, no significant cross-sectional association was found between epigenetic age acceleration and the diagnosis of diabetes. On the other hand, longitudinal analysis indicated that in male diabetic participants, each additional year of 7-CpG DNAm acceleration at baseline was associated with an 11% increased likelihood of developing new diabetes-related complications or exacerbating pre-existing complications during the follow-up period. A separate longitudinal study conducted among twins (27) found a positive correlation between blood glucose levels and epigenetic age markers. As this study is cross-sectional, it did not perform longitudinal comparisons, suggesting that future research should explore the relationship between epigenetic age and diabetes in more depth through longitudinal studies. Furthermore, due to sample limitations in the database, diabetes was not categorized in this study, highlighting the need for more detailed research with categorized data and larger sample sizes in future investigations.

### 4.2 The relationship between other epigenetic materials and diseases

In this study, epigenetic markers such as GDF15Mort, B2MMort, CystatinCMort, TIMP1Mort, ADMMort, PACKYRSMort, GrimAgeMort, GrimAge2Mort, HorvathTelo, DunedinPoAm, CD4TPP, and NeuPP were significantly higher in cancer patients compared to non-cancer patients (*p* < 0.05). These biomarkers are involved in key cellular processes, such as cell cycle regulation, inflammatory response, apoptosis, and gene expression regulation, all of which play crucial roles in cancer development and progression. GDF15 promotes tumor cell invasion and metastasis by regulating cell migration and apoptosis (28). High expression of GDF15 in various cancer types is associated with tumor invasiveness and poor prognosis (29). TIMP1 as a matrix metalloproteinase inhibitor, TIMP1 regulates matrix remodeling in the tumor microenvironment and promotes tumor metastasis (30, 31). Elevated levels of TIMP1 are linked to poor prognosis in multiple cancers (32–34).

### 4.3 Diabetes risk assessment model

This study employed machine learning techniques to analyze the relationship between 30 epigenetic biomarkers and the risks of diabetes and cancer, and it visualized the association between the most influential biomarkers and the outcomes. In the diabetes risk assessment model, the top three most important features identified were logA1Mort, the household income to poverty ratio, and marital status. Among these, logA1Mort demonstrated a linear relationship with the onset of diabetes, where its increase was positively correlated with an elevated risk of diabetes. LogA1Mort refers to the log-transformed glycated hemoglobin value derived from DNA methylation (35), and evidence indicates that it is strongly correlated with insulin, triglyceride, and glucose levels, as well as with insulin sensitivity and glucose metabolism (35). Importantly, studies have shown that for each standard deviation increase in logA1Mort, the risk ratio for coronary heart disease rises by approximately 30% (35). These findings suggest that logA1Mort could serve as a valuable biomarker for predicting diabetes, particularly in the context of an aging population. Integrating age with epigenetic biomarkers could facilitate the identification of high-risk groups, enabling earlier interventions. This study is the first to quantify the contribution of logA1Mort in diabetes prediction using machine learning techniques. By incorporating machine learning models alongside 30 epigenetic biomarkers, we were able to more accurately assess the contribution of these biomarkers to diabetes risk, offering a novel approach for early diabetes screening.

In this study, a poorer economic status, as indicated by a household income-to-poverty ratio close to 0 or 1, was associated with a higher likelihood of developing diabetes. When the household income-to-poverty ratio ranged from 1 to 4, the partial dependence values stabilized, showing no significant fluctuations in diabetes risk. However, when the poverty ratio exceeded 4, the partial dependence values sharply decreased, suggesting that a higher poverty ratio (which reflects better economic status) may be linked to a reduced risk of developing diabetes. The household income-to-poverty ratio, as a proxy for socioeconomic status, has been demonstrated in several studies to significantly influence both the incidence and prognosis of diabetes (36). This study further highlights the predictive role of income and poverty ratio in assessing diabetes risk, particularly in low-income groups, where both the incidence and mortality rates of diabetes are higher. Socioeconomic factors can elevate the risk of diabetes by influencing behaviors related to health, access to healthcare, and other factors. Therefore, future diabetes prevention and intervention strategies should take these socioeconomic variables into account, with a particular focus on enhancing early screening and health education for low-income populations.

In this study, marital status is also one of the important features in the diabetes model. Studies have pointed out (37) that for minority men and non-Hispanic white men, divorce/separation status is significantly associated with diabetes mortality, while for minority women and non-Hispanic white women, widowhood status is significantly associated with diabetes mortality. Some studies have shown (38, 39) that the impact of marital status on diabetes mortality differs by gender.

### 4.4 Cancer risk assessment model

In the cancer risk assessment model, gender, non-Hispanic White ethnicity, and PACKYRSMort were identified as the top three important features. Among these, gender was found to be one of the most significant contributing factors, which aligns with existing literature, underscoring gender as a critical element in cancer risk prediction. The incidence of various cancers shows notable differences between males and females. Several studies have indicated (40) that men generally exhibit a higher incidence of various cancer types compared to women, particularly cancers that are strongly linked to smoking and alcohol consumption, such as lung cancer, esophageal cancer, and metastatic cancers. Due to differences in endocrine functions, immune system responses, and other factors such as physical activity, men tend to have higher susceptibility to these cancers. For instance, in the case of lung cancer (41), men face a significantly higher risk compared to women, especially among smokers. Although smoking rates have decreased among women, the incidence of lung cancer in women has risen in recent years, which may be attributed to factors such as women’s smoking levels, genetic predispositions, and gender-specific differences in smoking behaviors. Conversely, breast cancer incidence is substantially higher in women than in men (42, 43), a difference closely tied to the role of estrogen in the female body. Estrogen promotes the development and lactation of breast tissue, and prolonged exposure to this hormone may increase the risk of developing breast cancer.

There is substantial evidence that habitual smoking is a major risk factor for immune-mediated inflammatory diseases and various cancers (44). Specifically, smoking is a major risk factor for head and neck cancers and lung cancer (45). Approximately 90% of lung cancer cases are directly related to smoking (46). Smoking is also closely linked to bladder cancer, pancreatic cancer, and renal pelvic cancer. Smoking is also associated with the etiology of colon cancer, liver cancer, and stomach cancer (45, 46). Existing data clearly indicate that smoking increases the risk of cancer in multiple organs (47) and often leads to premature death. The five-year survival rate for lung cancer is very low, only 15%. Other smoking-related cancers, such as liver cancer and pancreatic cancer, also have low five-year survival rates (18 and 9%, respectively). PACKYRSMort is a composite biomarker for estimating pack-years of smoking, proposed by Ake T. Lu based on DNA methylation (17). Our study shows that PACKYRSMort has a nonlinear relationship with cancer risk. When the cumulative smoking burden is <10, the risk of cancer is relatively low. When the cumulative smoking burden is between 10 and 30, the risk of cancer significantly increases. When the cumulative smoking burden is >30, the risk of cancer begins to decline. Our results differ from other studies. Rota et al. (48) quantified the dose–response relationship between smoking and gastric cancer risk and found that smoking is not only an independent risk factor for gastric cancer, but the risk increases with the number of cigarettes smoked daily, from a small amount to 20, and the risk increases in a dose-dependent manner with the duration of smoking. A meta-analysis by Lugo et al. showed that the risk of gallbladder cancer increases linearly with smoking intensity and duration (49). In addition, the risk of breast cancer (50) and cervical cancer (51) has also been reported to be linearly associated with smoking intensity. Our study is consistent with previous research when the cumulative smoking burden is ≤30, but when the cumulative smoking burden is >30, cancer risk shows a certain degree of decline. This phenomenon requires further biological explanation. A possible explanation is that the carcinogenic risk brought by long-term smoking has pushed the individual’s health status to the limit, and other health factors (such as immune decline, organ failure, etc.) may begin to significantly affect their cancer risk. Additionally, long-term smoking may alter DNA methylation patterns (52, 53), leading to the suppression or activation of certain gene expressions, thus affecting the mechanisms of cancer development.

In the cancer prediction model, the non-Hispanic White population emerged as a key sociodemographic factor, underscoring the significant role of race and cultural background in cancer risk prediction. Previous studies have shown (54) that, compared to non-Hispanic White individuals, Hispanic men and women experienced 25–30% lower cancer incidence (2014–2018) and mortality rates (2015–2019), with a notably lower incidence of common cancers. This study further substantiates the relevance of this group in the cancer model, highlighting the potential influence of racial differences on cancer susceptibility. These findings not only enhance our understanding of cancer risk prediction but also lay the groundwork for future personalized risk assessments. By integrating epigenetic biomarkers (such as PACKYRSMort) with sociodemographic factors (such as gender and non-Hispanic White ethnicity), we can more accurately identify high-risk populations, particularly in contexts where gender, race, and socioeconomic factors intersect. These results offer both theoretical support for personalized cancer prevention strategies and data-driven insights that can inform public health policy development.

## 5 Limitations and future directions

Although this study has made significant progress in exploring the relationship between epigenetic biomarkers and the risk of diabetes and cancer, and has successfully developed relevant predictive models, several limitations remain. These limitations highlight important areas for improvement and provide a clear direction for future research.

Firstly, although this study is based on a large amount of epigenetic data, the data is sourced from a single NHANES database. The types of diabetes and cancer are not further categorized, and the data is geographically limited to North America. Additionally, there are missing data points within the dataset, leading to sample imbalances. While standardization and other balancing techniques were applied during data processing, some bias remains inevitable. Therefore, future validation with external cohorts and additional cohort studies would be helpful for further analysis. Expanding data collection and analysis to include diverse regions, races, and cultural backgrounds would improve the model’s applicability and external validity.

Secondly, this study mainly relies on existing epigenetic clocks and machine learning techniques for risk prediction. While these methods have demonstrated their effectiveness in certain aspects, there is still significant potential for improvement in terms of algorithm complexity and predictive accuracy. For instance, training machine learning models requires large volumes of high-quality data, and challenges such as reducing data bias, addressing data loss, and improving feature selection accuracy remain critical areas for improvement. Additionally, the interpretability of these models is another key concern, particularly in the medical field, where it is essential to understand and explain the model’s predictions for clinical applications. Therefore, future research should place greater emphasis on enhancing the interpretability of these models, with a particular focus on integrating biological contexts to clarify the roles of various epigenetic biomarkers in disease prediction.

Thirdly, while epigenetic biomarkers offer promising new biological indicators for early regulation and risk assessment of diabetes and cancer, the genetic mechanisms involved are highly complex and operate across multiple regulatory levels. Single markers, such as specific DNA methylation sites or epigenetic age clocks, may not fully capture the underlying biological processes. To address this limitation, future research should incorporate multidimensional analyses that combine epigenetic biomarkers with other molecular markers, such as genetic mutations, proteomics, and transcriptomics. This multi-omics approach would provide a more comprehensive understanding of an individual’s aging process and enhance the accuracy of disease risk predictions.

Additionally, as a cross-sectional study, this research is limited in its ability to establish causal relationships and may be influenced by age-related effects or selection bias. To overcome these limitations, future research should consider conducting longitudinal studies to track changes in epigenetic biomarkers over time. This approach would help clarify causal relationships between epigenetic changes and disease development, providing a more dynamic understanding of how these biomarkers contribute to the progression of diseases such as diabetes and cancer.

## 6 Conclusion

This study is the first to combine epigenetic age acceleration with risk assessment of diabetes and cancer, and conducted a comprehensive analysis using machine learning models. The results indicate that epigenetic age acceleration is closely associated with cancer risk, but has a weaker relationship with diabetes. Through machine learning methods, the top three contributing features in the diabetes model are logA1Mort, household income-to-poverty ratio, and marital status, with an increase in logA1Mort being positively correlated with diabetes risk. In the cancer model, the top three contributing features are gender, non-Hispanic White ethnicity, and PACKYRSMort, with PACKYRSMort showing a nonlinear relationship with cancer risk.

## Data Availability

Publicly available datasets were analyzed in this study. The data can be accessed from the NHANES Database at: https://www.cdc.gov/nchs/nhanes/index.htm.
